# Anti-müllerian Hormone for the Prediction of Ovarian Response in Progestin-Primed Ovarian Stimulation Protocol for IVF

**DOI:** 10.3389/fendo.2019.00325

**Published:** 2019-05-28

**Authors:** Jialyu Huang, Jiaying Lin, Hongyuan Gao, Yun Wang, Xiuxian Zhu, Xuefeng Lu, Bian Wang, Xinyan Fan, Renfei Cai, Yanping Kuang

**Affiliations:** Department of Assisted Reproduction, Shanghai Ninth People's Hospital, Shanghai Jiao Tong University School of Medicine, Shanghai, China

**Keywords:** Anti-Müllerian hormone, ovarian response, progestin-primed ovarian stimulation, pregnancy, freeze-all strategy

## Abstract

**Background:** The ability of anti-Müllerian hormone (AMH) to predict ovarian response has been studied extensively in gonadotropin-releasing hormone agonist and antagonist treatments, but no information is available regarding its value in progestin-primed ovarian stimulation (PPOS) protocol.

**Methods:** This retrospective data analysis included 523 patients without polycystic ovary syndrome who underwent their first *in vitro* fertilization/intracytoplasmic sperm injection cycle with PPOS protocol at our center between Jan. 2015 and Jul. 2018. Serum AMH measurements were acquired within 12 months prior to ovarian stimulation using the automated Access AMH assay.

**Results:** AMH exhibited a significantly positive correlation with the number of retrieved oocytes (*r* = 0.744, *P* < 0.001). For the prediction of poor (<4 oocytes) and high (>15 oocytes) response, AMH had an area under the receiver operating characteristic curve (AUC) of 0.861 and 0.773, corresponding with an optimal cutoff point of 1.26 and 4.34 ng/mL, respectively. When stratified according to the dose of medroxyprogesterone acetate (MPA) (4 mg vs. 10 mg per day), AMH retained its similarly high predictive value for poor (AUC = 0.829 and 0.886, respectively) and high response (AUC = 0.770 and 0.814, respectively) in both groups. Amongst the 314 women who received their first frozen embryo transfer (FET) following PPOS protocol, no significant differences were observed on the rates of biochemical pregnancy, clinical pregnancy, implantation, early miscarriage, multiple pregnancy and ectopic pregnancy (all *P* > 0.05) across AMH quartiles (≤1.43, 1.44-2.55, 2.56–4.35, >4.35 ng/mL). In a multivariable logistic regression model, age was suggested to be the only independent risk factor for clinical pregnancy (*P* = 0.011).

**Conclusions:** Our data demonstrated that AMH is an adequate predictor of both high and poor ovarian response in PPOS protocol regardless of MPA dose, but it does not associate with pregnancy outcomes in the first FET cycles in a freeze-all strategy.

## Introduction

The optimization and individualization of controlled ovarian stimulation (COS) for *in vitro* fertilization (IVF) depends on utilizing patient characteristics and biomarkers to accurately predict ovarian response and tailor intended treatment. The characteristics, such as age, body mass index (BMI), menstrual cycle length, and results from previous IVF cycles are generally considered by clinicians for selection of ovarian stimulation strategies (1). In addition, several different markers of ovarian reserve, which usually refers to the number of available primordial follicles as well as the oocyte quality, have been proposed as predictors of ovarian response with varying degrees of success (2, 3). Of these, biochemical measures, such as basal follicle-stimulating hormone (FSH), estradiol (E_2_) and inhibin concentrations, fluctuate substantially during the menstrual cycle and hence their use has been limited (4, 5). Ovarian imaging, particularly antral follicle count (AFC), is largely affected by sonographers' intra- and inter-observer reproducibility and its sensitivity may differ from the resolution of transvaginal ultrasonography equipment (2, 6).

Anti-Müllerian hormone (AMH), a dimeric glycoprotein and a member of the transforming growth factor-β (TGF-β) family, has recently been demonstrated to be a promising surrogate marker of functional ovarian follicle reserve (5, 7). Produced by granulosa cells of preantral and small antral follicles, it acts as a follicular gatekeeper inhibiting initial follicle recruitment and FSH-dependent growth and selection (5). Unlike other ovarian reserve biomarkers, AMH has shown its superiority for good intra- and inter-cycle stability and good measurement repeatability (4, 5). Previous studies have extensively investigated the value of AMH in predicting both high and poor response in either gonadotropin-releasing hormone (GnRH) agonist or GnRH antagonist protocols (8–15), and the efficiency of an AMH-tailored stimulation regimen (1, 16, 17). However, so far no consensus on cutoff points of AMH has been achieved for ovarian response prediction as different COS protocols are inconsistent in the endocrine profile, early follicle recruitment and synchronization of follicular development, consequently resulting in a difference in the amount of oocytes retrieved (18). Besides, the method of AMH measurements in different clinical settings should be taken into consideration. For instance, the AMH concentrations detected by the Diagnostic System Laboratories assay have been reported to be 30% lower than those measured by the Gen I immunoassay (19). In addition, ethnicity has been associated with altered levels of AMH, with Chinese, Black African, Hispanic and South Asian women reported as having lower AMH than Caucasian women (20). Therefore, the predictive models based on AMH cannot be extrapolated directly from one ethnic population to another.

Recently, we reported a new COS protocol named progestin-primed ovarian stimulation (PPOS), in which medroxyprogesterone acetate (MPA), adjuvant to human menopausal gonadotropin (hMG), is used from the early follicular phase as an effective oral alternative to GnRH analogs for the prevention of premature luteinizing hormone (LH) surges during COS (21, 22). Based on the freeze-all policy, the PPOS protocol yields similar amount of oocytes and pregnancy outcomes compared with conventional short protocol in normal ovulatory women undergoing IVF/intracytoplasmic sperm injection (ICSI) (21). Subsequent studies have also proven its efficacy in women with poor ovarian response (23), polycystic ovarian syndrome (PCOS) (24) and advanced ovarian endometriosis (25), and demonstrated its safety in IVF newborns regarding neonatal outcome and congenital malformations (26). However, unlike the direct action on pituitary GnRH receptor in GnRH agonist and antagonist treatment, this new protocol was initially proposed for the consideration that administration of exogenous progestin (P) could inhibit GnRH/LH surge via the P receptor in the hypothalamus and block the E_2_-induced positive feedback effects (22, 27). Therefore, differences have been noted between PPOS protocol and other conventional regimens, including the total gonadotropin dose and endocrine changes during COS (21, 23, 24).

The question therefore remains whether AMH can predict ovarian response to PPOS protocol at a level of accuracy comparable to that of GnRH agonist and antagonist treatment. Moreover, given the dearth of evidence concerning the predictive role of AMH among Chinese women undergoing IVF (11, 12, 14), and the controversy on whether AMH has any correlation with IVF outcomes (15, 28–32), the current study attempted to establish the predictive value of AMH in ovarian response and assess the relationship between serum AMH and pregnancy outcomes in IVF using PPOS protocol.

## Materials and Methods

### Study Population and Design

The present work was a retrospective analysis of a cohort study performed at the Department of Assisted Reproduction of Shanghai Ninth People's Hospital affiliated with Shanghai Jiao Tong University School of Medicine. Our study protocol was approved by the hospital's Ethics Committee (Institutional Review Board) (No: 2014–31). We selected patients with measured AMH levels within the previous 12 months before the COS started from January 2015 to July 2018. This time interval has been proven with reliable consistency regarding the predictive value of AMH (33). The inclusion was limited to patients with a regular cycle who underwent their first IVF/ICSI cycle with PPOS protocol regardless of age. Patients were excluded from the study if they met one of the following criteria: (1) diagnosis of PCOS in accordance with the modified Rotterdam diagnostic criteria (34); (2) documented history of ovarian surgery (i.e., laparoscopic ovarian drilling, ovarian endometrioma stripping ,and unilateral oophorectomy); (3) use of hormonal contraceptives for pretreatment before the study cycle; (4) core data missing in the medical records (e.g., without endometrial thickness on the day of embryo transfer).

### Endocrine Assays and AFC Measurement

Basal serum concentrations of FSH, LH, E_2_, and P were analyzed on menstrual cycle day 3 (MC3) before the start of stimulation using chemiluminescence (Abbott Biologicals B.V., the Netherlands). The analytical sensitivity was as follows: FSH, 0.06 IU/L; LH, 0.09 IU/L; E_2_, 10 pg/mL; and P, 0.1 ng/mL. We determined serum levels of AMH with the automated Access AMH assay (Beckman Coulter, Inc., USA). The assay's detection range was between 0.08-24 ng/mL with the detection limit of 0.02 ng/mL. Coefficients of variation were 1.5% (intra-assay) and 3.9% (inter-assay) for low (0.87 ng/mL), 1.4% (intra-assay) and 3.0% (inter-assay) for medium (4.45 ng/mL), and 1.7% (intra-assay), and 3.5% (inter-assay) for high (13.70 ng/mL) AMH levels. The AFC was detailed as the combined number of follicles with diameters between 2 and 10 mm in both ovaries as measured by transvaginal ultrasound scan on MC3.

### Ovarian Stimulation Protocol

A description of the PPOS protocol has been presented in detail in our previous publications (21, 35). Briefly, patients were administered with hMG (150 or 225 IU/d; Anhui Fengyuan Pharmaceutical Co., China) and MPA (4 or 10 mg/d; Shanghai Xinyi Pharmaceutical Co., China) from MC3 onward. The initiating dose was 150 IU/d for patients with high AFC (>20) and those with elevated basal FSH (>7 IU/L), while 225 IU/d was used for all other patients. Follicular monitoring, along with measurement of serum FSH, LH, E_2_, and P concentrations, were initiated on MC7-8 and performed every 2–4 days. The dose of hMG was adjusted depending on the growing follicles and E_2_ level during the stimulation. When the leading follicle reached 18 mm in diameter, the final stage of oocyte maturation was cotriggered using triptorelin (0.1 mg; Decapeptyl, Ferring Pharmaceuticals, Germany) and human chorionic gonadotropin (hCG) (1,000 IU; Lizhu Pharmaceutical Trading Co., China). Transvaginal ultrasound-guided oocyte retrieval was undertaken 34–36 h after trigger. All follicles with diameters over 10 mm were aspirated.

The aspirated oocytes were fertilized *in vitro* by either conventional insemination or ICSI according to semen parameters. The freeze-all strategy was performed for all IVF/ICSI cycles. According to the criteria described by Cummins et al. (36), only embryos classified as top-quality (grade I and II) were cryopreserved via vitrification on day 3 after oocyte retrieval, whereas embryos graded as quality III and IV were subjected to extended culture and observation up to the blastocyst stage. The Gardner and Schoolcraft grade system (37) was then applied to select blastocysts with good morphological grades (grade ≥3BC) for vitrification on day 5 or 6.

### Endometrium Preparation and Frozen Embryo Transfer

Endometrial preparation and frozen embryo transfer (FET) were performed as previously described (21). In short, FET was conducted in a natural cycle for patients with regular menstrual cycles, while patients with irregular menstrual cycles were treated with letrozole and, if necessary, in combination with hMG to stimulate monofollicular growth. Hormone replacement therapy (HRT) was recommended for patients with thin endometrium during either natural cycles or stimulated cycles. Up to two embryos per patient were transferred in each FET cycle. The transfer of day 3 or day 5–6 embryos was scheduled according to the timing of ovulation during the natural and mild stimulation cycle and the timing of P administration during HRT. Once a pregnancy was achieved, the luteal support was continued to 10 weeks of gestation.

### Outcome Measures

The primary outcome measure was to determine whether serum levels of AMH have any correlation with the number of oocytes retrieved. Furthermore, we aimed to evaluate the ability of AMH to successfully predict high and poor response, and investigate whether the predictive ability differed according to the MPA dose (4 or 10 mg/d) applied in PPOS protocol. The threshold for high response was set at >15 oocytes retrieved (8, 12, 13), while a poor response was defined as <4 retrieved oocytes or cycle cancellation in accordance with the Bologna criteria (9, 12, 13, 38). A normal response was therefore defined as 4–15 oocytes retrieved. The total cumulative dose of hMG and duration of stimulation were recorded, as well as the number of >10 mm and >14 mm follicles on trigger day, number of oocytes retrieved, number of metaphase II oocytes, number of fertilized oocytes, number of two pronuclei (2PN) oocytes and number of embryos available.

In the second part of our study, we attempted to assess the association between AMH concentration and pregnancy outcomes among patients who received their first FET before May 2018. Clinical pregnancy was defined as the presence of a gestational sac regardless of the presence or absence of fetal heart activity, as measured by ultrasound examination 7 weeks after FET. The implantation rate was defined as the number of gestational sacs divided by the number of embryos transferred. The early miscarriage rate was defined as the percentage of patients with spontaneous pregnancy termination prior to the gestational age of 12 weeks.

### Statistical Analysis

Statistical analysis was performed using the SPSS (version 20.0; SPSS Inc., USA), MedCalc (version 15.0; MedCalc Software bvba, Belgium) and STATA (version 12.0; StataCorp LLC, USA). Because none of the continuous data studied showed normal distribution under both Kolmogorov-Smirnova and Shapiro-Wilk test, they were presented as median with interquartile range, while categorical data was presented as frequencies with percentages. Between-group statistical differences were assessed by Kruskal-Wallis test and Chi-square test or Fisher's exact test for continuous and categorical variables, respectively. The correlation between baseline variables and the number of oocytes retrieved was evaluated using Spearman's rank correlation coefficients (*r*). Variables correlated were further included in a multivariate linear regression model to identify the independent determinants related to the number of retrieved oocytes. Receiver operating characteristic (ROC) curves were generated for each of the selected parameters to determine their ability to predict high or poor ovarian response. To assess differences in the predictive ability of AMH in the context of utilized MPA dose (4 mg/d vs. 10 mg/d) in PPOS protocol, the areas under the curves (AUCs) were compared using the method described by DeLong et al. (39). Optimal cutoff points were determined by the combination of specificity and sensitivity closest to the optimal.

For the comparison of pregnancy outcomes, patients were grouped based on AMH quartiles: ≤ 25th (≤ 1.43 ng/mL), 25–50th (1.44–2.55 ng/mL), 50–75th (2.56–4.35 ng/mL), >75th (>4.35 ng/mL). A multivariable logistic regression was further performed to investigate the effect of potential risk factors on clinical pregnancy. Potential risk factors, including age, BMI, AMH, MPA dose during COS, number of embryos transferred, endometrial preparation and endometrial thickness on FET day, were introduced into the regression equation by the forward stepwise (likelihood ratio) method. Unadjusted and adjusted odds ratios (ORs) and 95% confidence intervals (CIs) were calculated by the regression models. The −2 log likelihood was used to determine the significance of the models, and the Nagelkerke's R^2^ was used to evaluate and explain uncertainty. All *P-*values were based on two-sided tests and *P* <0.05 was considered to be statistically significant.

## Results

### Baseline Characteristics, Stimulation Characteristics and Outcomes According to Ovarian Response

Overall, 523 women who underwent the PPOS treatment protocol for their first IVF/ICSI cycle were included in the study. High ovarian response was observed in 77 (14.7%) women, while 182 (34.8%) were categorized as poor responders. The patients' baseline characteristics according to the level of ovarian response were shown in Table 1. The three groups differed significantly in age, basal FSH, basal E_2_, AMH and AFC (all *P* <0.01). However, no significant differences were found when BMI, subfertility type, duration and causes, basal LH and basal P were analyzed.

**Table 1:** Baseline characteristics according to the type of ovarian response.

	**High response (oocytes >15)**	**Normal response (4≤ oocytes ≥15)**	**Poor response (oocytes <4)**	***P-*value**
No. of patients	77	264	182	
**Demographics**				
Age (years)	30.0 (28.0–32.5)	33.0 (30.0–37.0)	36.5 (32.0–40.0)	<0.001
BMI (kg/m^2^)	21.6 (19.9–24.0)	21.1 (19.5–23.0)	21.5 (19.5–23.4)	0.353
**Fertility characteristics**				
Primary subfertility, *n* (%)	33 (42.9)	125 (47.3)	84 (46.2)	0.785
Secondary subfertility, *n* (%)	44 (57.1)	139 (52.7)	98 (53.8)	
Duration of subfertility (years)	2 (1–4)	2 (1–4)	2 (1–4)	0.965
**Cause of subfertility**, ***n*** **(%)**				
Tubal factor	45 (58.0)	136 (51.5)	92 (50.5)	0.375
Male factor	8 (10.4)	27 (10.2)	15 (8.2)	
Endometriosis	0 (0.0)	17 (6.4)	14 (7.7)	
Unexplained	0 (0.0)	2 (0.8)	3 (1.6)	
Mixed/other	24 (31.2)	82 (31.1)	58 (31.9)	
**Endocrinological profile**				
Basal FSH (IU/L)	5.16 (4.39–5.78)	5.05 (5.80–6.52)	7.70 (6.04–10.36)	<0.001
Basal LH (IU/L)	3.37 (2.79–4.79)	3.19 (2.34–3.96)	3.18 (2.28–4.26)	0.486
Basal E_2_ (pg/mL)	31.0 (23.0–39.0)	34.5 (27.0–44.0)	37.5 (26.0–49.0)	0.009
Basal P (ng/mL)	0.3 (0.2–0.4)	0.3 (0.2–0.4)	0.3 (0.2–0.4)	0.460
AMH (ng/mL)	5.38 (3.48–7.38)	2.79 (1.67–4.34)	0.76 (0.42–1.50)	<0.001
Antral follicle count	16 (12–20)	9 (7–13)	5 (3–7)	<0.001

*Data are presented as median (interquartile range) for all continuous variables*.

Table 2 demonstrates the stimulation characteristics and outcomes per started cycle. The proportions of patients in different ovarian response categories who received the hMG + MPA (4 mg/d) protocol statistically significantly decreased across the three groups (*P* <0.001), from 64 (83.1%) in the high response group to 85 (46.7%) in the poor response group. The opposite trend was observed for the hMG + MPA (10 mg/d) protocol applied. The poor-responding patients had received a significantly lower dose of gonadotropin compared with the normal-responding and high-responding patients (*P* <0.001), while the duration of stimulation was similar among groups (*P* = 0.117). There was a significant between-group difference for the cycle stimulation outcomes, including the number of >10 mm and >14 mm follicles on trigger day, number of oocytes retrieved, number of metaphase II oocytes, number of fertilized oocytes, number of 2PN oocytes as well as number of embryos available (all *P* <0.001).

**Table 2:** Stimulation characteristics and outcomes according to the type of ovarian response.

	**High response (*n* = 77)**	**Normal response (*n* = 264)**	**Poor response (*n* = 182)**	***P-*value**
**Stimulation characteristics**				
hMG + MPA (4mg/d), *n* (%)	64 (83.1)	202 (76.5)	85 (46.7)	<0.001
hMG + MPA (10mg/d), *n* (%)	13 (16.9)	62 (23.5)	97 (53.3)	
Total dose of hMG (IU)	2025 (1800–2250)	2025 (1800–2250)	1575 (1125–1950)	<0.001
Duration of stimulation (days)	9 (9–10)	9 (8–10)	8 (7–9)	0.117
**Stimulation outcomes**				
No. of >10 mm follicles on trigger day	21.0 (17.0–26.5)	11.0 (8.0–14.0)	3.0 (2.0–5.0)	<0.001
No. of >14 mm follicles on trigger day	15.0 (11.0–20.0)	7.0 (5.0–10.0)	2.0 (1.0–4.0)	<0.001
No. of oocytes retrieved	19.0 (17.0–22.5)	9.0 (6.0–12.0)	2.0 (1.0–3.0)	<0.001
No. of metaphase II oocytes	17.0 (14.0–20.0)	8.0 (6.0–10.0)	2.0 (1.0–3.0)	<0.001
No. of fertilized oocytes	14.0 (12.0–18.0)	6.5 (5.0–9.0)	2.0 (1.0–3.0)	<0.001
No. of 2PN oocytes	12.0 (9.5–14.0)	5.0 (4.0–7.0)	1.0 (1.0–2.0)	<0.001
No. of embryos available	8.0 (3.5–9.0)	3.0 (2.0–5.0)	1.0 (0.0–2.0)	<0.001

*Data are presented as median (interquartile range) for all continuous variables*.

### Predictive Ability of AMH for Ovarian Response

The level of AMH exhibited a strong positive correlation with the number of oocytes retrieved according to Spearman's rank correlation analysis (*r* = 0.744, *P* < 0.001) (Supplementary Figure 1A). A significant but weaker correlation was also shown between AFC, basal FSH, age, basal E_2_ and oocyte yield (*r* = 0.740, *P* < 0.001; *r* = −0.552, *P* < 0.001; *r* = −0.394, *P* < 0.001; and *r* = −0.122, *P* = 0.005, respectively), while no significant correlation was observed with regard to BMI (*r* = −0.025, *P* = 0.565) (Supplementary Figures 1B–F). After construction of a multivariate linear regression model, the largest influencing independent factor for the number of retrieved oocytes was AFC, followed by AMH, age, MPA dose and basal FSH in order of decreasing importance (Table 3). No significant association was observed between total hMG dose and oocyte yield (*P* = 0.806).

**Table 3:** Multiple linear regression analysis of possible determinants for number of oocytes retrieved.

**Independent variables**	**Unstandardized coefficients**		**Standardized coefficients**		***P-*value**
	**β (95% CI)**	**Std. Error**	**β**	***t***	
(Constant)	7.528 (3.840 to –11.216)	1.877	-	4.010	<0.001
AFC	0.465 (0.364 to –0.566)	0.051	0.370	9.041	<0.001
AMH (ng/mL)	0.926 (0.723 to –1.130)	0.104	0.346	8.924	<0.001
Age (years)	−0.142 (−0.219 to –0.064)	0.040	−0.110	−3.582	<0.001
MPA dose (10 vs. 4 mg)	−1.210 (−2.129 to –0.290)	0.468	−0.082	−2.585	0.010
Basal FSH (IU/L)	−0.147 (−0.281 to –0.012)	0.068	−0.076	−2.143	0.033
Total hMG dose (IU)	0 (−0.001 to –0.001)	0	0.008	0.246	0.806

*The model (R = 0.763, R^2^ = 0.583, adjusted R^2^ = 0.578, P < 0.001). The values of the standardized coefficients reflect the independent contributions of each predictor to dependent variables*.

**Figure 1:**
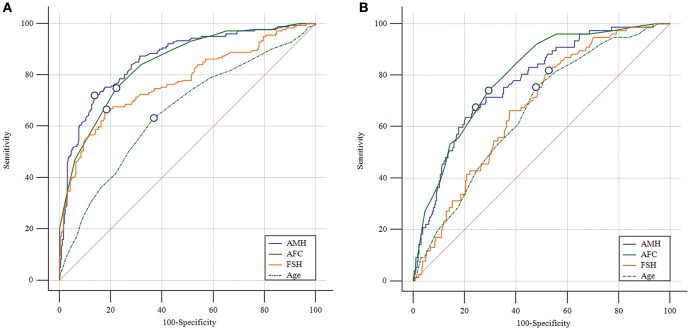
Receiver operating characteristic curves for AMH, AFC, basal FSH and age for ovarian response prediction. **(A)** Prediction of poor (<4 oocytes) response. **(B)** Prediction of high (>15 oocytes) response. The marked point is in correspondence with Youden index.

The predictive abilities of AMH, AFC, age and basal FSH for ovarian response were further analyzed by ROC curves (Figure 1). AMH showed a high accuracy for the prediction of both poor and high response with an AUC of 0.861 (95% CI: 0.825–0.892) and 0.773 (95% CI: 0.725–0.817), respectively. The AMH cutoff value for poor response prediction was 1.26 ng/mL with a sensitivity of 72.0% and a specificity of 86.4%, while the threshold of 4.34 ng/mL was shown to predict high response with a sensitivity of 67.5% and a specificity of 75.8%. The AUC values of AFC were comparable to those of AMH for prediction of poor and high response (AUC = 0.843 [95% CI: 0.806–0.876] and 0.797 [95% CI: 0.751–0.839]; *P*_AMHvs.AFC_ = 0.374 and 0.420, respectively). Basal FSH and age, however, performed significantly worse than AMH. The AUC values of basal FSH for poor and high response were 0.773 (95% CI: 0.731–0.811; *P*_AMHvs.FSH_ = 0.001) and 0.673 (95% CI: 0.621–0.723; *P*_AMHvs.FSH_ = 0.021), and those of age were 0.656 (95% CI: 0.609–0.700; *P*_AMHvs.FSH_ < 0.001) and 0.659 (95% CI: 0.606–0.710; *P*_AMHvs.age_ < 0.001), respectively.

**Figure 2:**
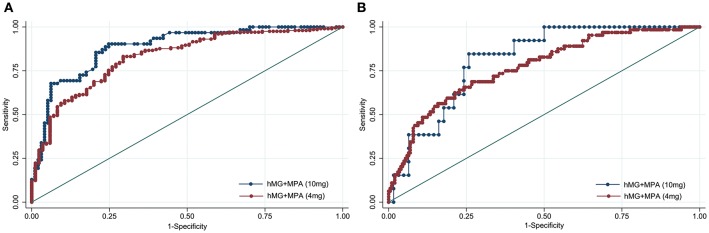
Receiver operating characteristic curves for AMH for ovarian response prediction according to the MPA dose. **(A)** Prediction of poor (<4 oocytes) response. **(B)** Prediction of high (>15 oocytes) response. The diagonal line is the reference line of no discrimination (area under the curve = 0.5).

To investigate whether the predictive ability of AMH was affected by the MPA dose applied in PPOS treatment, ROC curves were constructed for poor and high response accordingly (Figure 2). The curves revealed that the AUC values of AMH were comparable between hMG + MPA (4 mg/d) and hMG + MPA (10 mg/d) protocol: 0.829 (95% CI: 0.778–0.880) vs. 0.886 (95% CI: 0.834–0.981) for poor response, *P* = 0.125; and 0.770 (95% CI: 0.704-0.835) vs. 0.814 (95% CI: 0.709–0.919) for high response, *P* = 0.485.

### Pregnancy Outcomes According to AMH Quartiles

A total of 314 women (60.0%) undergoing FET were stratified according to the 25, 50, and 75th percentiles of the serum AMH concentration. Due to the significant difference in number of embryos available for transfer across AMH quartiles (*P* < 0.001) (Table 4), only the first FET cycles were included for analysis. No significant differences, however, were observed among the AMH quartiles for all the analyzed pregnancy parameters, including biochemical pregnancy rate (*P* = 0.084), clinical pregnancy rate (*P* = 0.158), implantation rate (*P* = 0.144), early miscarriage rate (*P* = 0.346), multiple pregnancy rate (*P* = 0.132) and ectopic pregnancy rate (*P* = 0.278), as detailed in Table 4.

**Table 4:** Pregnancy outcomes of the first FET cycle according to the AMH level.

	**AMH quartiles (ng/mL)**				***P-*value**
	**≤1.43**	**1.44–2.55**	**2.56–4.35**	**>4.35**	
No. of patients	79	79	78	78	
No. of viable embryos per patient	2 (1–3)	3 (2–4)	4 (3–6)	4 (3–8)	<0.001
AMH (ng/mL)	0.79 (0.46–1.04)	2.01 (1.68–2.23)	3.28 (2.92–3.83)	6.31 (5.19–7.72)	<0.001
**Demographics**					
Age (years)	37.0 (33.0–40.0)	34.0 (30.0–38.0)	33.0 (29.0–37.0)	31.0 (28.0–34.3)	<0.001
BMI (kg/m^2^)	21.0 (19.5–23.4)	20.8 (19.5–23.1)	21.0 (19.4–23.0)	21.9 (20.1–23.8)	0.634
**FET characteristics**					
Total No. of transferred embryos	122	142	145	143	
Single embryo transfer, *n* (%)	36 (45.6)	16 (20.3)	11 (14.1)	13 (16.7)	<0.001
Double embryo transfer, *n* (%)	43 (54.4)	63 (79.7)	67 (85.9)	65 (83.3)	
**Endometrial preparation**					
Natural cycle, *n* (%)	17 (21.5)	15 (19.0)	17 (21.8)	8 (10.3)	0.016
HRT, *n* (%)	24 (30.4)	16 (20.3)	9 (11.5)	13 (16.7)	
Mild stimulation, *n* (%)	38 (48.1)	48 (60.8)	52 (66.7)	57 (73.1)	
Endometrial thickness (mm)	9.8 (8.5–11.3)	9.5 (8.3–11.3)	10.1 (8.4–11.2)	10.6 (9.5–12.5)	0.096
**Pregnancy outcome**, ***n/N*** **(%)**					
Biochemical pregnancy rate	30/79 (38.0)	40/79 (56.0)	45/78 (57.7)	35/78 (44.9)	0.084
Clinical pregnancy rate	27/79 (34.2)	39/79 (49.4)	38/78 (48.7)	31/78 (39.7)	0.158
Implantation rate	34/122 (27.9)	48/142 (33.8)	54/145 (37.2)	37/143 (25.9)	0.144
Early miscarriage rate	5/27 (18.5)	5/39 (12.8)	2/38 (5.3)	5/31 (16.1)	0.346
Multiple pregnancy rate	7/27 (25.9)	7/39 (17.9)	15/38 (39.5)	6/31 (19.4)	0.132
Ectopic pregnancy rate	0/27 (0.0)	2/39 (5.1)	0/38 (0.0)	2/31 (6.5)	0.278

*Data are presented as median (interquartile range) for all continuous variables*.

Unadjusted and adjusted ORs and 95% CIs of the potential risk factors for clinical pregnancy are shown in Table 5. Age and number of embryos transferred were significantly related to clinical pregnancy in unadjusted analysis (*P* = 0.010 and *P* = 0.042, respectively). In adjusted analysis, the only independent variable was found to be age (*P* = 0.011). Women ≥41 years had a significantly lower incidence of clinical pregnancy than women <30 years (OR = 0.27, 95% CI: 0.10–0.80).

**Table 5:** Crude and adjusted odds ratios of confounding factors for clinical pregnancy in the first FET cycle.

**Variables**	**Crude OR (95% CI)**	***P-*value**	**Adjusted OR (95% CI)**	***P-*value**
Age (years)		0.010		0.011
<30	Reference		Reference	
30–34	1.46 (0.80–2.66)		1.51 (0.82–2.78)	
35–37	1.66 (0.81–3.40)		1.72 (0.84–3.53)	
38–40	1.06 (0.50–2.23)		1.04 (0.49–2.21)	
≥41	0.26 (0.09–0.74)		0.27 (0.10–0.80)	
BMI (kg/m^2^)		0.569		–
<18.5	0.82 (0.39–1.72)		-	
18.5–24.9	Reference		-	
≥25	0.70 (0.35–1.42)		-	
AMH (ng/mL)		0.161		-
≤ 1.43	0.55 (0.29–1.04)		-	
1.44–2.55	1.03 (0.55–1.92)			
2.56–4.35	Reference		-	
>4.35	0.69 (0.37–1.31)		-	
MPA dose during COS (mg)		0.939		-
4	Reference		-	
10	1.02 (0.62–1.67)		-	
No. of embryos transferred		0.042		-
1	Reference		-	
2	1.75 (1.02–3.02)		-	
Endometrial preparation		0.478		-
Natural cycle	Reference		-	
HRT	0.85 (0.42–1.75)		-	
Mild stimulation	0.71 (0.39–1.28)		-	
Endometrial thickness (mm)		0.514		-
<8	0.71 (0.35–1.45)		-	
8–11	Reference		-	
>11	1.10 (0.67–1.80)		-	

*Adjusted ORs were adjusted for all covariates in the table using a binary logistic regression model through forward stepwise method. The −2 log likelihood = 405.58, and the Nagelkerke R^2^ = 0.069*.

## Discussion

The results of the study provided evidence for the first time that AMH as a single test is adequately predictive of both high and poor ovarian response in patients undergoing PPOS protocol for IVF. This predictive ability is unaltered by the different dose of MPA applied in PPOS treatment. Furthermore, our study found no significant association between AMH level and pregnancy outcomes in the first FET cycles in a freeze-all strategy.

The findings from the current study are in line with previous researches on the high predictive value of AMH for ovarian response using either GnRH agonist or antagonist protocols (8–15). Our data revealed that both AMH and AFC are better predictors of ovarian response during COS compared with other traditional measures (i.e., age and basal FSH level). These two markers of ovarian reserve exhibit comparable predictive value for ovarian response in PPOS protocol, in accordance with previous studies indicating that early-follicular phase AFC and AMH have similar correlations to the number of oocytes retrieved (3, 6, 40). Direct comparisons of AFC and AMH in ovarian response prediction have generally shown no significant difference, while a few studies demonstrated that AMH or AFC had stronger predictive value than the other (6). Since each method has its own advantages and drawbacks (2, 6), a combination of both could potentially be used to assess the ovarian reserve comprehensively, although AMH has been found to be a better predictor of oocyte yield in patients with discordant AFC and AMH measurements (41).

PPOS protocol is established based on the inhibitory effects of P on pulsatile GnRH and pituitary LH and FSH discharges, as well as its prevention of E_2_-induced positive feedback effects (21, 27). The current study found that MPA 4 mg/d was preferentially used in patients with high response, while MPA 10 mg/d was applied more frequently for poor responders at our center. This is mainly based on the hypothesis that a higher dose of MPA could lead to a deeper pituitary suppression and prevent spontaneous ovulatory LH surge more effectively, especially for women of advanced age, diminished ovarian reserve, and elevated basal LH levels (42). However, a recent prospective randomized controlled trial (RCT) has demonstrated comparable endocrinological characteristics and clinical outcome of PPOS protocol using different doses of MPA (35). The ROC analysis in our study also revealed that the predictive values of AMH for both high and poor response remain constant irrespective of MPA dose, further strengthening that the administration of 4 mg of MPA daily is sufficient for a desirable outcome in women undergoing IVF/ICSI treatment (35).

The cutoff level of AMH should be interpreted with caution and assessed by the evaluation of eventual benefits vs. the possible misclassification of patients. A threshold of 4.34 ng/mL is set for high response in PPOS protocol, which implies an elevated risk of ovarian hyperstimulation syndrome (OHSS) for patients above this level and a need for more intense monitoring of ovarian stimulation. However, coupled with dual trigger (GnRH agonist and a low dose of hCG) for final oocyte maturation and the application of a freeze-all strategy for viable embryos, PPOS protocol allows for nearly complete avoidance of the incidence of OHSS (21, 43). Regarding poor response, the AMH cutoff value is 1.26 ng/mL with a sensitivity of 72.0% and a specificity of 86.4%. Patients with AMH below this threshold should be informed in advance of their relatively low opportunity of achieving pregnancy due to a significantly higher rate of no available embryos (36.9 vs. 7.3%, *P* <0.001). Nevertheless, it should not be used in isolation as the criterion for withholding fertility treatment (30, 31). Through repeated COS cycles, it is rational to assume an increased cumulative pregnancy rate since the developmental potential of embryos showed no difference between AMH below and above 1.26 ng/mL, as indicated by the similar clinical pregnancy rate following their first FET cycle (39.1 vs. 44.1%, *P* = 0.463).

Accurate prediction of ovarian response is of paramount importance in individualized gonadotropin dose selection (1). Previous cohort studies have shown that AMH-tailored stimulation strategies resulted in a decreased incidence of high and poor response, increased pregnancy and live birth rates, as well as a reduction in costs (16, 17). These findings, however, are challenged by two recent RCTs to some extent (44, 45). In the single-center study by Allegra et al. (44), no significant differences were observed in the clinical pregnancy rate or the number of embryos cryopreserved per patient between FSH starting dose selection based on a nomogram (age, day 3 FSH and AMH) and an age-based strategy, despite a significant increase in the proportion of patients with optimal ovarian response. Another multicenter RCT of 1329 women further demonstrated that individualized FSH dosing based on serum AMH and body weight was non-inferior for ongoing pregnancy and implantation rates as well as the risk of moderate to severe OHSS (45). In our study, patients of poor response require significantly lower dose of gonadotropin than those of normal and high response, in contrast with the higher gonadotrophin dose needed for maximal stimulation in poor responders undergoing long GnRH agonist protocol (10, 12). One potential explanation may be the mechanism that the inhibitory action of P on the GnRH/LH surge is mediated by the classical P nuclear receptor of the hypothalamus rather than pituitary GnRH receptor, and by blockade of the activation and transmission of the E_2_-induced signal (27). Therefore, unlike the pituitary desensitization in long agonist protocol, PPOS protocol exhibited an indirect, mild and slow suppression of LH secretion through continuous administration of MPA (21), leading to a lower dose of gonadotropin for stimulating growth and development of fewer follicles. This finding would lay a foundation for future design of prospective well powered studies on the efficacy and safety of different dosing regimens in PPOS protocol determined by an individual's AMH level.

Given that serum AMH concentration correlates strongly with oocyte yield, it is plausible that AMH might also be associated with qualitative outcomes of ovarian stimulation. Several large-scale retrospective analyses have shown a positive association between AMH and implantation, pregnancy and live birth rates after assisted reproduction (15, 29), with the confirmation from a prospective cohort study even after adjusting for age and oocyte yield (28). However, others have found no such association (30, 31). Due to the conflicting results of accumulating data, a meta-analysis of 19 studies has been carried out recently which suggested that AMH has a weak correlation with implantation and clinical pregnancy but its predictive accuracy is limited (32). To date, this is the first study to demonstrate no significant differences in pregnancy outcomes in the first FET cycles across AMH quartiles in a freeze-all strategy. Instead, age serves as the only risk factor for clinical pregnancy, which is easy to understand since increased age is well-characterized by a reduction in both oocyte quantity and quality and accompanied with a decline in female fecundity (46). Thus, AMH may be less promising in predicting pregnancy chances of women undergoing IVF, although further prospective studies are still awaited.

A major weakness of the current study stems from its retrospective and non-randomized design, although the ascertainment and recall bias were minimized because all the data were gathered and documented in the computerized database. Also, there was no attempt to compare the ability of AMH in ovarian response prediction between PPOS and other conventional COS protocols. Since PPOS protocol is the prior and mainstream COS regimen at our center, and previous studies have extensively investigated the predictive role of AMH in GnRH agonist and GnRH antagonist treatment (11, 12, 14), we therefore decided not to make a direct comparison in this study. Finally, analysis of the association between AMH and pregnancy outcomes was limited in the first FET cycle, without assessing the rates of cumulative clinical pregnancy and live births. Considering that no trial has been published regarding the predictive value of AMH for pregnancy in the freeze-all policy and that FET equals or even surpasses fresh embryo transfer on clinical outcomes following IVF (47), it is essential and vital for further research in this field.

## Conclusion

Our study demonstrates that AMH is an adequate predictor of both high and poor ovarian response in PPOS protocol, independent of the dose of MPA. However, AMH does not correlate with pregnancy outcomes in the first FET cycles in a freeze-all strategy. Therefore, to render infertility counseling and care more tailored to the patient, AMH level should be determined before embarking on PPOS treatment. Further studies are urgently needed to investigate the efficiency, safety and cost-effectiveness of individualized gonadotropin dosing based on the AMH level prior to IVF.

## Ethics Statement

The present work was a retrospective analysis of a cohort study performed at the Department of Assisted Reproduction of Shanghai Ninth People's Hospital affiliated with Shanghai Jiao Tong University School of Medicine. Our study protocol was approved by the hospital's Ethics Committee (Institutional Review Board) (No: 2014-31).

## Author Contributions

JH, RC, and YK contributed to the conception and design of the study. JL, HG, YW, XZ, and XL were responsible for data collection and checking. JH performed the data analysis, interpretation and manuscript drafting. BW and XF were involved in data visualization. RC and YK supervised the project administration. All authors read and approved the final manuscript.

### Conflict of Interest Statement

The authors declare that the research was conducted in the absence of any commercial or financial relationships that could be construed as a potential conflict of interest.
