# Supplementary material for: Anti-müllerian Hormone for the Prediction of Ovarian Response in Progestin-Primed Ovarian Stimulation Protocol for IVF
Source: Front Endocrinol (Lausanne). 2019 May 28;10:325. doi: 10.3389/fendo.2019.00325 (PMC6547790; doi:10.3389/fendo.2019.00325)
Supplement: Supplementary file 1 [file Image_1.pdf]

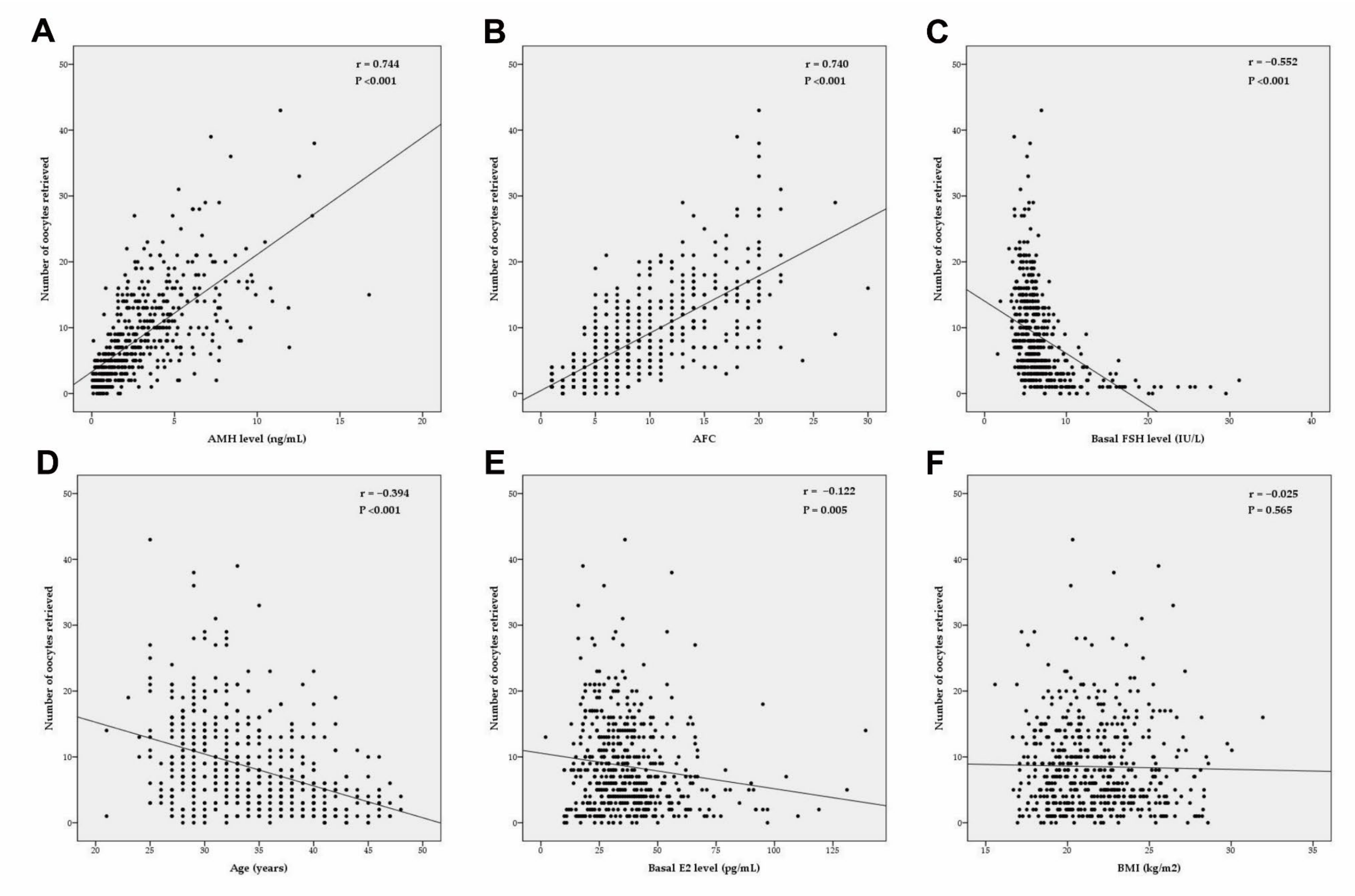

Supplementary figure 1. Correlation analysis between patients' baseline characteristics and the number of oocytes retrieved. (A) AMH level, (B) AFC, (C) basal FSH level, (D) age, (E) basal E_2 level, and (F) BMI. The Spearman's rank correlation coefficients (*r*) and *P* value are shown in the top right. The linear line is the line of best fit.
